# Risk Analysis of Air Pollution and Meteorological Factors Affecting the Incidence of Diabetes in the Elderly Population in Northern China

**DOI:** 10.1155/2020/3673980

**Published:** 2020-10-20

**Authors:** Yao Lin, Saijun Zhou, Hongyan Liu, Zhuang Cui, Fang Hou, Siyuan Feng, Yourui Zhang, Hao Liu, Chunlan Lu, Pei Yu

**Affiliations:** ^1^NHC Key Laboratory of Hormones and Development, Tianjin Key Laboratory of Metabolic Diseases, Chu Hsien-I Memorial Hospital & Tianjin Institute of Endocrinology, Tianjin Medical University, Tianjin 300134, China; ^2^Department of Epidemiology and Health Statistics, Tianjin Medical University, Heping District, Tianjin, China; ^3^Community Health Service Center, Jiefang Road, Tanggu Street, Binhai New District, Tianjin, China

## Abstract

**Background:**

Research investigating the effect of air pollution on diabetes incidence is mostly conducted in Europe and the United States and often produces conflicting results. The link between meteorological factors and diabetes incidence remains to be explored. We aimed to explore associations between air pollution and diabetes incidence and to estimate the nonlinear and lag effects of meteorological factors on diabetes incidence.

**Methods:**

Our study included 19,000 people aged ≥60 years from the Binhai New District without diabetes at baseline. The generalized additive model (GAM) and the distributed lag nonlinear model (DLNM) were used to explore the effect of air pollutants and meteorological factors on the incidence of diabetes. In the model combining the GAM and DLNM, the impact of each factor (delayed by 30 days) was first observed separately to select statistically significant factors, which were then incorporated into the final multivariate model. The association between air pollution and the incidence of diabetes was assessed in subgroups based on age, sex, and body mass index (BMI).

**Results:**

We found that cumulative RRs for diabetes incidence were 1.026 (1.011-1.040), 1.019 (1.012-1.026), and 1.051 (1.019-1.083) per 10 *μ*g/m^3^ increase in PM_2.5_, PM_10_, and NO_2_, respectively, as well as 1.156 (1.058-1.264) per 1 mg/m^3^ increase in CO in a single-pollutant model. Increased temperature, excessive humidity or dryness, and shortened sunshine duration were positively correlated with the incidence of diabetes in single-factor models. After adjusting for temperature, humidity, and sunshine, the risk of diabetes increased by 9.2% (95% confidence interval (CI):2.1%-16.8%) per 10 *μ*g/m^3^ increase in PM_2.5_. We also found that women, the elderly (≥75 years), and obese subjects were more susceptible to the effect of PM_2.5_.

**Conclusion:**

Our data suggest that PM_2.5_ is positively correlated with the incidence of diabetes in the elderly, and the relationship between various meteorological factors and diabetes in the elderly is nonlinear.

## 1. Introduction

Diabetes is one of the top ten reasons for increases in Years Lived with Disability (YLD) and exacerbates the global burden of disease [1]. More than 80% of diabetes deaths occur in low- and middle-income countries, and China has the highest diabetes prevalence [2, 3]. Therefore, it is essential to predict diabetes incidence accurately and to control its possible risk factors. It has been widely recognized that family history, age, obesity, and genetic susceptibility are all risk factors for diabetes, and recent evidence supports the hypothesis that air pollution might be an essential risk factor for diabetes. Since most risk factors are uncontrollable, air pollution acting as an intervening factor may provide a turning point for the prevention of diabetes and reduce the heavy burden caused by this condition [4–8].

Suspended particulate matter (PM) and gaseous pollutants such as ozone (O_3_), nitrogen dioxide (NO_2_), carbon monoxide (CO), and sulfur dioxide (SO_2_) are major air pollutants posing a threat to human health. Previous research has shown that exposure to PM_2.5_ may increase the risk of diabetes or death due to diabetes [9–11]. Possible mechanisms behind this increased risk include oxidative stress, systemic inflammation, immune response, insulin resistance, disorders of the autonomic nervous system, and alterations of mitochondrial function and gene expression in adipose tissue [12]. Air pollution possibly acts through pathways that affect glucose homeostasis, thereby increasing the risk of diabetes [13–15].

In addition to air pollutants, meteorological factors are also closely related to human health, and the effects of climate change have a disproportionate impact on the health of vulnerable groups in low- and middle-income countries [16]. There are close connections between meteorological factors and noncommunicable diseases, such as cardiovascular, respiratory, and metabolic diseases [17–19]. A few of these studies explored the association between meteorological factors and diabetes and found that extreme temperatures increase diabetes mortality and hospitalization rates and affect glucose homeostasis. Additionally, sunshine can reduce the incidence of diabetes [20–23]. However, studies on other meteorological factors, such as humidity, wind speed, and precipitation, are rare.

Although evidence of air pollution, especially PM, is increasing, diabetes-related results to guide the establishment of air quality standards remain controversial. Furthermore, most studies are conducted in developed countries, and there is a lack of evidence from developing countries in Asia. However, the estimated prevalence of diabetes among adults in China was 10.9% in 2013 [24]. The prevalence was higher in rural Tianjin than in the overall Chinese rural and urban population, according to the Fourth National Nutritional Survey [25]. China is a developing country with the largest population in the world. With rapid urbanization and industrialization, the burning of large amounts of fuel and the emission of vehicle exhausts have resulted in decreased air quality [26]. The most polluted cities are mainly located in North China Plain, which includes Tianjin [27]. With the implementation of the Air Pollution Prevention Action Plan, which was the most stringent air pollution plan to date in China, the concentrations of SO_2_, NO_2_, PM_10_, and PM_2.5_ decreased in Tianjin from 2012 to 2017, but still exceeded the national standard air quality index (AQI) level II and far exceeded the WHO guidelines. Air pollution caused significant public health problems, affecting over 100 million people in the Beijing-Tianjin-Hebei (BTH) region [28]. Given the heavy burden of diabetes and the inevitable exposure to air pollution, the association between air pollution and diabetes may have clinical and public health significance, especially in Tianjin, China. Meteorological factors are also risk factors for diabetes, and there are interactions or synergies between meteorological factors and air pollutants [29]. However, studies about the impact of meteorological factors on diabetes incidence are limited, and results are inconsistent due to differences in geographic locations and races studied. Therefore, an evaluation of the relationship between meteorological factors and diabetes is urgently warranted. The purpose of this study was to investigate the effects of air pollutants and meteorological factors on the incidence of diabetes to provide theoretical support and reference for environmental management as well as to relieve the burden of diabetes.

## 2. Material and Methods

### 2.1. Study Population and Area

The Binhai New District (east longitude 117.68, north latitude 39.03) is located in the Eastern coastal area of Tianjin. It is the center of the Bohai Economic Circle, with a total area of 2,270 square kilometers and a population of 2.97 million. We included residents aged 60 years and older who regularly participated in free medical examinations in Tianjin Binhai New District. A total of 37 community hospitals were involved, covering approximately 360,000 people. After screening the data, subjects with diabetes at baseline, residents under the age of 60, and residents with repeated physical examinations within one year were excluded. This resulted in a total of 190,453 people included in this study.

### 2.2. Health Data

The daily incidence of diabetes in Tianjin Binhai New District from January 1, 2014, to December 31, 2017, was obtained from the Tianjin Community Health Service Information System (http://218.68.9.122:808/), an official website for collecting and registering basic information and medical examination data for residents aged ≥60 years in Tianjin. Residents from 37 community hospitals in Tianjin Binhai New District participated for free in the annual medical examinations. In addition to the date of diagnosis and incidence of diabetes, the trained doctors also collected biochemical test values, including triglycerides, cholesterol, serum urea nitrogen, serum creatinine, aspartate aminotransferase, alanine aminotransferase, fasting blood glucose, hemoglobin, platelets, and white blood cells. The following demographic characteristics and behavioral health data were also collected by a face-to-face conversation on the day of medical examinations: gender, age, weight, height, body mass index (BMI), smoking status, drinking status, and exercise frequency. Finally, the above information was electronically recorded. The diseases in this study were coded according to The International Classification of Diseases, Revision 10 (ICD-10). The crude rate of diabetes diagnosed in community hospitals was 3.7 per 1,000 person-years, and additional patients were diagnosed in higher-level hospitals. Finally, the data were screened by ID card number, a unique number for national legal identity, to ensure that there were no duplications.

### 2.3. Diagnosis of Diabetes

We defined a new-onset diabetes event as a patient who was without diabetes on January 1, 2014, and then diagnosed with diabetes during the study. Diabetes was diagnosed based on the criteria of the WHO Diabetes Committee in 1999. Diagnosis of diabetes included the following: diagnosed as diabetes by medical personnel or self-reported diagnosis of diabetes in hospital (ICD-10 code: E10-E14); taking antidiabetic drugs or subcutaneous insulin therapy within the 12 months before the medical examination; or diagnosed by medical personnel and recommended for diabetes diet and physical activity programs. Although we cannot rule out type 1 diabetes, considering that on average >90% of adult diabetes is type 2 diabetes and our research was aimed at elderly aged ≥60 years, it can be assumed that most cases were type 2 diabetes [30]. All subjects who met the above diagnostic criteria for diabetes at baseline (January 1, 2014) were considered preexisting diabetes patients and were excluded from the study.

### 2.4. Air Pollution Data

According to the National Environmental Protection Standard of the People's Republic of China (http://www.mee.gov.cn/GB3095-2012), real-time air quality is measured every hour by the four Air Quality Monitoring (AQM) stations located in Binhai New District and data of particulate pollutants (PM_2.5_ and PM_10_) and gaseous pollutants (NO_2_, SO_2_, CO, and O_3_) are recorded. We downloaded data of air pollutants from January 1, 2014, until December 31, 2017, from the website of the Tianjin Ecological Environment Monitoring Center (http://www.tjemc.org.cn/index.html) and calculated the arithmetic mean of air pollutants concentrations for every 24 hours, except for O_3_, which was calculated as a daily 8-hour maximum O_3_ concentration, as the daily average concentration of air pollutants at each monitoring point. An average of these four monitoring stations' concentrations of air pollutants was used as the daily average concentration of air pollutants in Binhai New District. The average concentration of air pollutants within a day was calculated only when there were more than 20 valid data. Before calculating the average concentrations, the hourly data of each station were checked for soundness to remove the nonconforming data by the “is.na” function.

### 2.5. Meteorological Data

All meteorological factors were measured eight times per day, at local time of 02, 05, 08, 11, 14, 17, 20, and 23 hours. These measurements were used to calculate the daily average. Meteorological data were downloaded from the China Meteorological Data Sharing Service System (http://data.cma.cn/), including daily average temperature (°C), relative humidity (%), wind speed (m/s), atmospheric pressure (hPa), precipitation (mm), and duration of sunshine (h). The average level (mean value) of four monitoring sites in Tianjin Binhai New District was used as the exposure level for residents.

### 2.6. Statistical Analysis

Correlations between air pollutants and meteorological factors were assessed by the Spearman rank correlation test, and the collinearity between independent variables was examined. Based on experience, the occurrence of diabetes in the population is a small probability event, and the overall distribution of cases is scattered, consistent with the Poisson distribution. Therefore, the Poisson's generalized additive model (GAM) was chosen as the model framework in this study. In view of the existing biological and epidemiological studies, we decided the confounding factors that need to be adjusted (day of the week (DOW), time, and meteorological factors) when focused on air pollution. Because the incidence of diabetes was not only affected by air pollution or meteorological factors of the day, but also related to the exposure levels of the previous few days. In order to fit the nonlinear and delayed effects, we used a distributed lag nonlinear model (DLNM) to construct a “cross-basis” function, a bidimensional function expressed by two basic functions, depicting the effects of predictors and lags simultaneously. The structure of our model for air pollution was as follows:

(1) $$\begin{array}{c} \text{LogE}\left(\text{Yt}\right)=\beta Z_{t,l}+\text{DOW}+\text{ns}\left(\text{time},\text{df}\right)+\text{ns}\left(\frac{\text{temperature}}{\cdots },\text{df}\right)+\text{intercept}, \end{array}$$

where Yt represented the expected number of diabetes cases; *βZ*_*t*,*l*_ represented the cross-basis objects used to estimate the effects of air pollutants; *Z* was determined by each pollutant (PM_2.5_, PM_10_, NO_2_, SO_2_, O_3_, and CO); *β* was the coefficient for *Z*_*t*,*l*_, the logarithmic increase in the number of diabetes cases caused by the increase of air pollutant by one unit; *t* represented the observation day, and *l* represented the lag days; ns was a natural cubic spline function, and df was its degree of freedom; time was a date variable used to control time trends and seasonal fluctuations; DOW represented the day of the week, controlling the natural fluctuations of the number of people with diabetes in a week; and temperature/⋯ referred to meteorological conditions, which were used to adjust the impact of meteorological factors on diabetes incidence. To fit the model, we set the df of natural cubic spline functions for time to 11 per year, which was based on the Akaike information criterion (AIC) and the principle of the partial autocorrelation function (PACF) [31]. And we set the df of meteorological factors to 3, based on experience and previous studies. The model for meteorological factors is shown in the Supplementary Materials (available here).

We first used a single-pollutant model to examine the association between the incidence of diabetes and the exposure of each air pollutant on the day (lag 0 days) to obtain estimated values that were not adjusted for meteorological factors. Additionally, the relationships between meteorological factors (lag 0 days) and diabetes incidence were analyzed. To verify the stability of the association between air pollution and diabetes incidence, a double-pollutant model was constructed. In addition, to avoid underestimating the relationship between air pollutants, meteorological factors, and diabetes, we studied the hysteresis and cumulative effects of air pollutants and meteorological factors on the incidence of diabetes. This allowed for the assessment of the impact of various factors on the occurrence of diabetes over time and the clarification of the duration of each factor's effect. Then, we constructed a three-dimensional model of meteorological factors and diabetes. Based on the single-factor models, statistically significant air pollutants (PM_2.5_, NO_2_, and CO) were included in the final multivariate model, and relevant meteorological factors (temperature, humidity, and sunshine) were selected as covariates. In the data processing process, we found that sixteen days' data of air pollutants in 2014 were missing, and the “na.approx” function from the “zoo” package in R software was used to fill the missing data with linear interpolated values [32].

We performed subgroup analyses of air pollutants, taking the sensitivity to air pollutants of different populations into account. We evaluated possible effect modification of these demographic characteristics, stratifying the population into different groups by sex (male or female), age (<75 or ≥75 years), and BMI (<18.5 kg/m^2^, 18.5-23.99 kg/m^2^, 24-27.9 kg/m^2^, and ≥28 kg/m^2^).

In all statistical tests, a two-sided *P* < 0.05 was considered significant. All analysis was performed in R software, version 3.5.1, and the fitting process was implemented using the “dlnm” and “mgcv” packages. The effect-estimated value is the relative risk ratio (RR) of the onset of diabetes and its 95% confidence interval (CI) for each 10 *μ*g/m^3^, 1 mg/m^3^, or interquartile range (IQR) increase of the corresponding air pollutant's concentration.

## 3. Results

**Figure 1:**
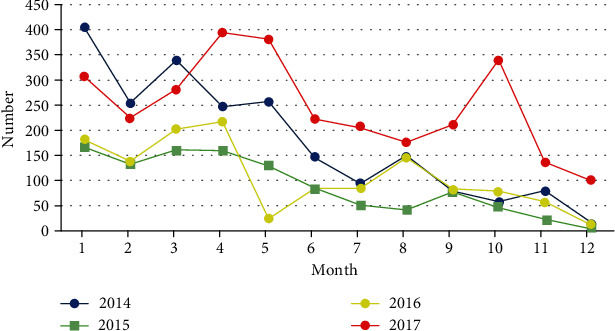
Monthly distribution of new-onset diabetes during 2014 to 2017.

Finally, 190,453 participants aged ≥60 years in Tianjin Binhai New District were included in the study, and the population characteristics are summarized in Table 1. The median (interquartile range) age was 68 (64-72) years, and the ratio of male to female was 1 : 1.09. A total of 49.31% of enrolled participants exercised every day, and 28.05% of participants never exercised. The participants did not have diabetes at baseline, and the median (interquartile range) value of fasting blood glucose (FBG) was 5.30 (4.91-5.70) mmol/L. During 2014-2017, there were 7,585 new-onset diabetes patients among the 190,453 nondiabetic participants, resulting in a crude incidence of diabetes of 9.9 per 1,000 person-years. There were 4,180 females (55%), 1,371 subjects aged ≥75 years (18%), and 5,413 overweight and obese people (71%) among these new-onset diabetes patients.  Figure 1 shows the monthly distribution of new-onset diabetes patients in the study from 2014 to 2017. The number of new-onset diabetes cases fluctuated to varying degrees every month. From 2014 to 2016, the number of new-onset diabetes had declined, but this rebounded in 2017. In general, diabetes was diagnosed more often in winter and spring (January, March to May), and less in summer (June to July).

Tables 2 and 3 show the statistical summary of air pollutants and meteorological factors. The average concentration of PM_2.5_ was 70.56 *μ*g/m^3^, which was more than once higher than the national standard (35 *μ*g/m^3^). The average concentrations of PM_10_, SO_2_, CO, O_3_, and NO_2_ were 96.24 *μ*g/m^3^, 23.55 *μ*g/m^3^, 1.42 mg/m^3^, 53.18 *μ*g/m^3^, and 48.2 *μ*g/m^3^, respectively. The corresponding median of the temperature, humidity, sunshine, air pressure, precipitation, and wind speed were 15.4°C, 57%, 8 h, 1016.6 hPa, 0 mm, and 2.3 m/s, respectively.

**Figure 2:**
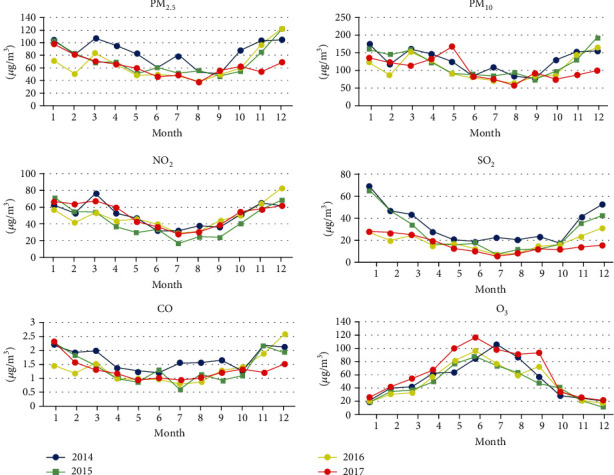
Monthly average changes in air pollutants for 2014-2017.

**Figure 3:**
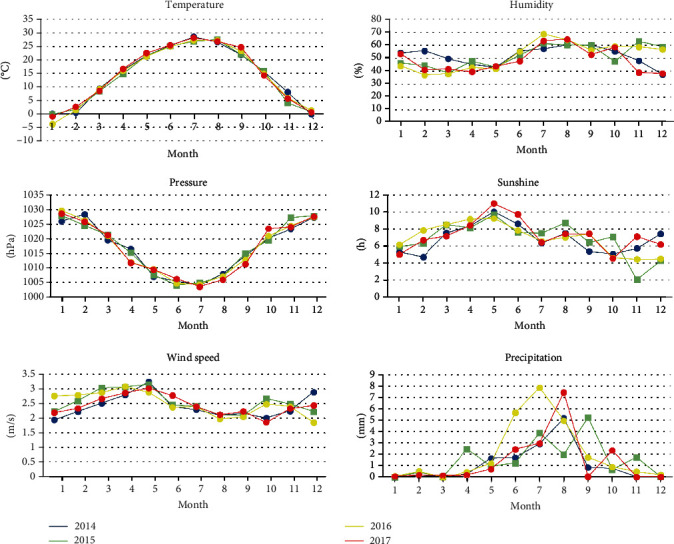
Monthly average changes in meteorological factors for 2014-2017.

Figures 2 and 3 show the monthly distribution of each air pollutant and meteorological factor from 2014 to 2017. The PM_2.5_, PM_10_, NO_2_, SO_2_, and CO all reached the highest concentration in winter (December-February) and the lowest in summer (June-August), while O_3_ levels were showing the opposite effect. Temperature and air pressure had apparent seasonal fluctuations, and the temperature was higher in summer and lower in winter, which was opposite to the air pressure. The regularity of humidity and precipitation was similar. Wind speed changed with no discernable patterns, but the wind speed was faster in spring (March-May).

Table 4 lists the results of the Spearman correlation between air pollutants and meteorological factors. PM_2.5_, PM_10_, NO_2_, SO_2_, and CO were positively correlated with each other, with correlation coefficients ranging from 0.548 to 0.897, while O_3_ was negatively correlated with all other air pollutants. O_3_ was positively correlated with all meteorological factors except for atmospheric pressure. Except for O_3_, all other pollutants were negatively correlated with sunshine duration and positively correlated with atmospheric pressure.

**Figure 4:**
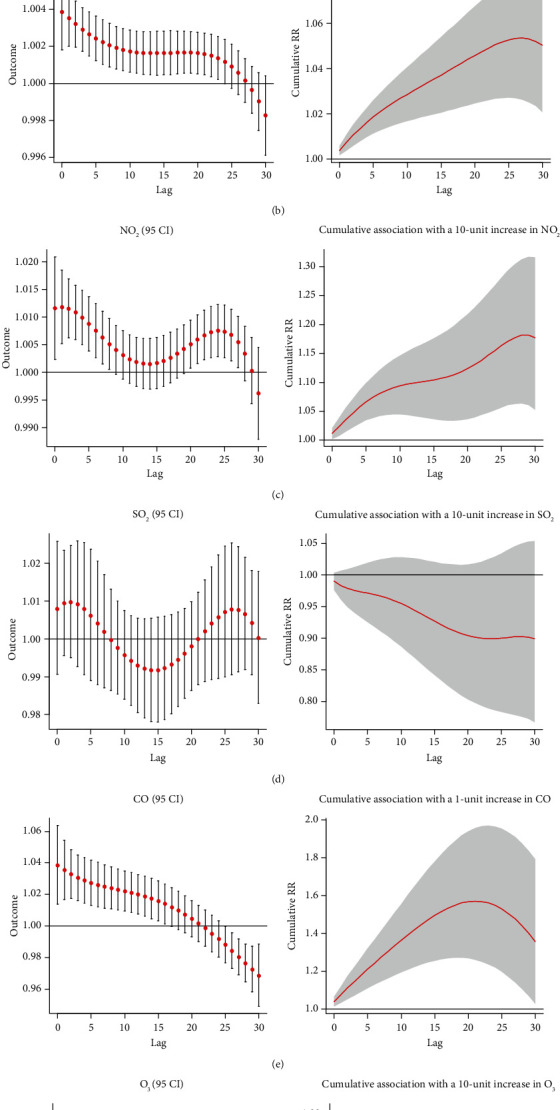
Hysteresis and cumulative effect of air pollutants on the incidence of diabetes in single-pollutant model ((a) PM_2.5_, (b) PM_10_, (c) NO_2_, (d) SO_2_, (e) CO, and (f) O_3_).

Figure 4 shows the delayed (longest lag 30 days) and the cumulative effect of the association between air pollutants and the onset of diabetes in a single-factor model.

- PM_2.5_: exposure to PM_2.5_ increased the incidence of diabetes, and the effect was the strongest on the day and then decayed gradually. By the 25th day, the effect was no longer present, and the cumulative effect also rose within 25 days (Figure 4(a)).
- PM_10_: the lag and cumulative effect of PM_10_ on diabetes incidence were similar to that of the PM_2.5_. The negative effect was strongest on the day and then gradually decayed until the 25th day. However, compared to PM_2.5_, PM_10_ had a weaker effect on the incidence of diabetes (Figure 4(b)).
- NO_2_: there was a positive correlation between NO_2_ levels and diabetes incidence within 8 days and 20-27 days. Although NO_2_ was also positively correlated with diabetes on days 9-19, the association was not statistically significant. Combined with the cumulative effect curve, exposure to NO_2_ was associated with an increased incidence of diabetes (Figure 4(c)).
- SO_2_: for both the single-effect and the cumulative-effect curves, no relationship between SO_2_ and diabetes incidence was found (Figure 4(d)).
- CO: exposure to CO is a relative risk factor for diabetes within 16 days, after which there was no significant correlation between CO and diabetes (Figure 4(e)).
- O_3_: in the single-effect curve, O_3_ was negatively correlated with the incidence of diabetes between the 12th day and the 23rd day. The cumulative-effect curve showed that O_3_ and the incidence of diabetes were negatively correlated from the 5th day (Figure 4(f)).

**Figure 5:**
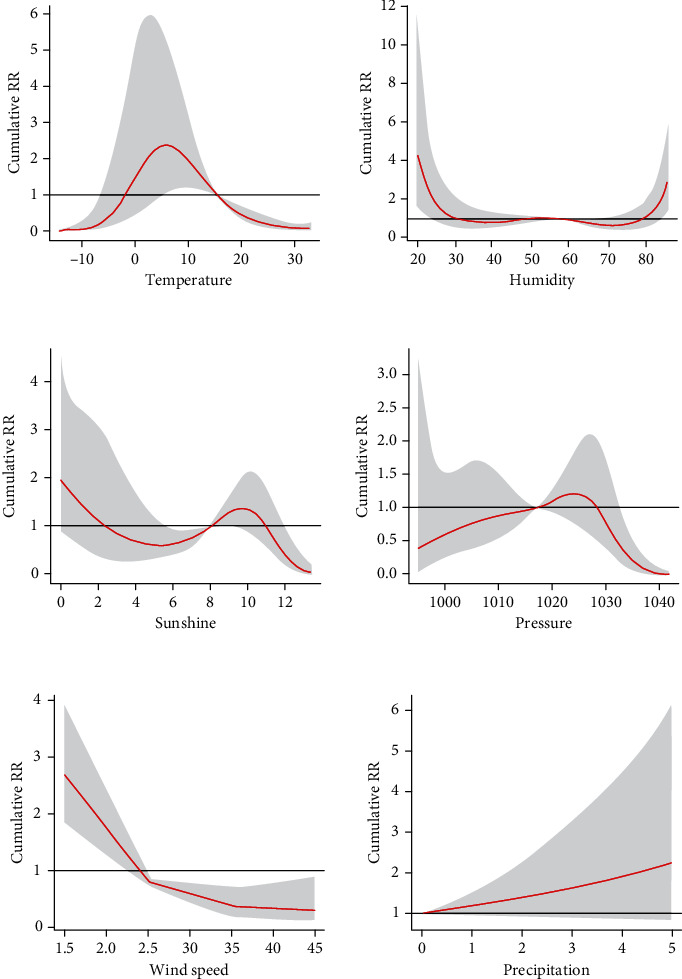
Cumulative effect curve of different meteorological factors on the incidence of diabetes.

**Figure 6:**
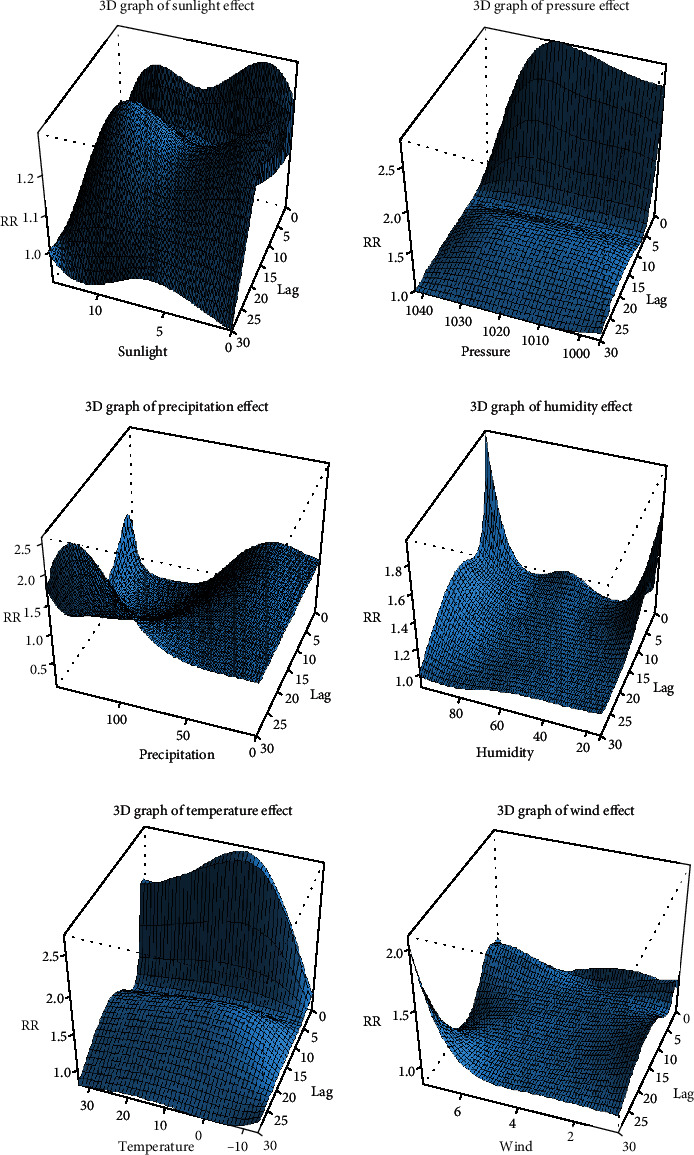
Three-dimensional image of the association between meteorological factors and the incidence of diabetes.

**Figure 7:**
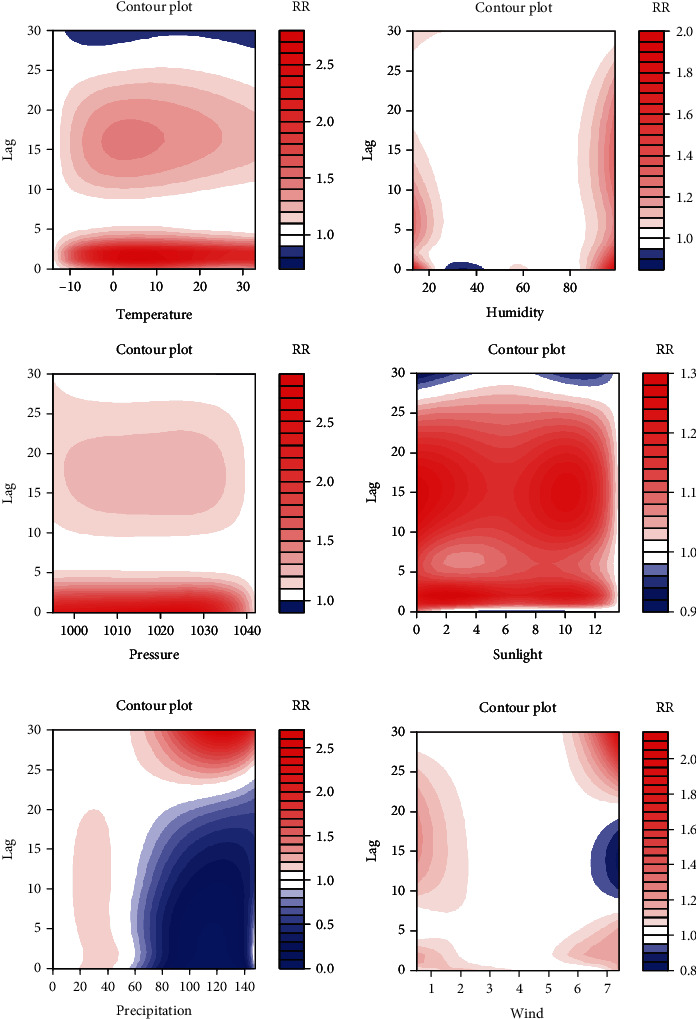
Contour plot of the relationship between meteorological factors and diabetes incidence.

Figure 5 shows the cumulative effect of lag 30 days for each meteorological factor in single meteorological factor models. The cumulative relative risks over 30 lag days of the full range of meteorological factors are presented. The black lines are the effect estimates, and the gray areas are the 95% CI. The median values were used as the reference for calculating the cumulative effect of various meteorological factors to study the lowest cumulative effect of different meteorological factors. The values of meteorological factors with the lowest cumulative effect value for 30 days were -14.5°C for temperature, 71% for humidity, 1042.7 hPa for atmospheric pressure, 13.6 h for the sunshine duration, 0 mm for precipitation, and 4.5 m/s for wind speed. These values were used as references to estimate the effects of meteorological factors on diabetes, as presented in Figures 6 and 7.

The relationship between meteorological factors and the incidence of diabetes was nonlinear.  Figure 6 shows three-dimensional (3D) graphs distributed in a hexahedron, which vividly depicts the relationship of meteorological factors, lag days, and risk of diabetes incidence (referenced to the values of meteorological factors with the lowest cumulative effect for 30 days). One bottom edge of the hexahedron represents the full range of meteorological factors, the other bottom edge represents the number of days delayed, and the height represents the relative risk of diabetes incidence. The 3D graphics show the impact of different meteorological factor values on the incidence of diabetes when lagging on different days.  Figure 7 shows contour plots of various meteorological factors depicting the estimated effects on diabetes incidence of the full range of meteorological factors over different lag days (referenced to the lowest effect value of meteorological factors). It is similar to the plot in Figure 6 and describes the risk of diabetes incidence with color. Blue indicates that the RR value was <1, and red indicates an RR value of >1. In other words, blue indicates a protective factor, and red indicates a risk factor, and the darker the red color, the higher the risk of diabetes.

- Humidity: whether excessively moist or dry, humidity increased the risk of diabetes, and the effect of moisture lasted longer. The humidity in the Binhai New District usually ranged from 43% to 72%, which meant that an adverse effect on the incidence of diabetes only occurred in extreme weather conditions.
- Sunshine: the shortening of sunshine duration was positively correlated with diabetes incidence, and the correlation was strongest within five days of exposure.
- Atmosphere pressure: the atmospheric pressure fluctuated between 994.2 and 1042.7 hPa. The risk of diabetes increased when the pressure was low, especially on the day. After the first five days, the effect was weakened but lasted for about 30 days.
- Precipitation: the risk of diabetes slightly increased when the precipitation reached a certain level. When precipitation further increased to 60 mm or more, it was negatively correlated with the risk of diabetes.
- Wind speed: wind speed ranged from 1.8 m/s to 3.0 m/s. A decrease in wind speed increased diabetes incidence, and the influence was more obvious within five days.

Table 5 summarizes the RR of diabetes in single and double-pollutant models for 10 *μ*g/m^3^ (1 mg/m^3^) or an interquartile range increase in air pollutant concentration. In the single-pollutant model, an increase in PM_2.5_, PM_10_, or NO_2_ concentrations by 10 *μ*g/m^3^ was associated with an increase in the risk of diabetes by 2.6%, 1.9%, and 5.1%, respectively. When the concentration of CO increased by 1 mg/m^3^, the risk of diabetes increased by 15.6%. The interquartile range increase in the concentrations of PM_2.5_, PM_10_, NO_2_, and CO contributed to an increased risk of diabetes by 14.4%, 15.7%, 17.1%, and 15.6%, respectively. The estimator of effect for both PM_10_ and PM_2.5_ was relatively robust in the dual-pollutant model. There was no statistical association between SO_2_ and O_3_ and the risk of diabetes in both the single-pollutant and the dual-pollutant models.

Based on the results of the single-factor model and experience from previous research, we selected relevant factors into the final multivariate model. The correlation between PM_2.5_ and PM_10_ was strong, with a correlation coefficient of 0.897. We considered there was collinearity between the two pollutants, and therefore, these parameters could not be included in the same final model. Similarly, the correlation coefficient of temperature and atmospheric pressure was -0.886. To avoid the influence of collinearity, atmospheric pressure was excluded from the final model. The correlation coefficients of the relation between SO_2_ and NO_2_ or CO were >0.7, and since SO_2_ had no apparent correlation with diabetes, we excluded it. Finally, PM_2.5_, NO_2_, and CO were included in the multivariate model. The diffusion of air pollutants was usually affected by meteorological factors. Therefore, we added temperature, humidity, and duration of sunshine to the final model as covariates to correct for these confounding factors.

**Figure 8:**
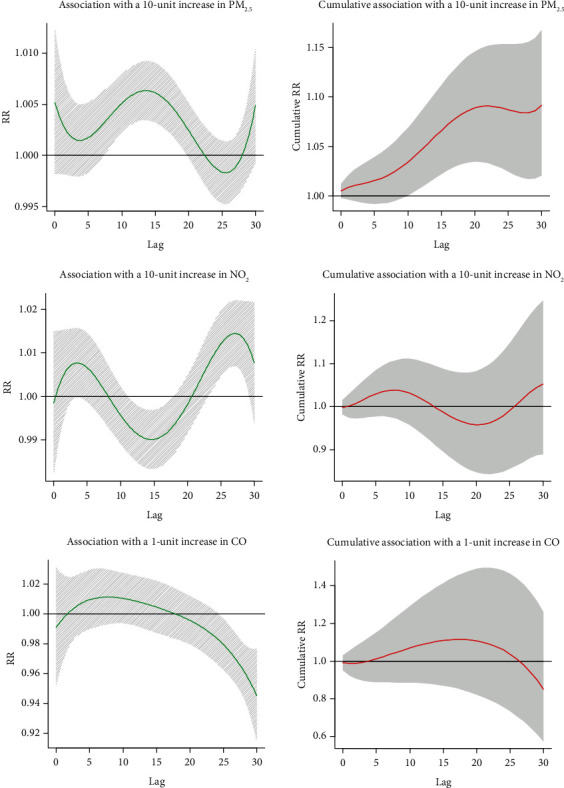
Hysteresis and cumulative effect of air pollutants on diabetes in multivariate model.

Separate and cumulative effect curves are shown in Figure 8. PM_2.5_ showed a positive correlation with the incidence of diabetes after eight days, and the adverse effect continued for more than 30 days. The cumulative RR of 30 days was 1.092 (95% CI: 1.021-1.168). In the separate effect curve, there was no significant correlation between NO_2_ and the risk of diabetes in the first 11 days, while NO_2_ was the protective factor for diabetes on the 11th-17th day. After that, NO_2_ became a risk factor of diabetes between the 23rd and 29th days. The correlation was not evident, and the cumulative effect curve showed no statistically significant correlation between NO_2_ and diabetes risk (RR: 1.053; 95% CI: 0.889-1.247). Overall, in the final model adjusted for PM_2.5_, NO_2_ was not significantly associated with the onset of diabetes. Finally, combining the separate effect curves and cumulative effect curves, the relationship between CO and diabetes incidence was not statistically significant in the final model (RR: 0.851; 95% CI: 0.574-1.261).

The modification effects of age, gender, and BMI on the relationship between air pollution and diabetes incidence were explored by grouping the study population based on these parameters (Table 6). We found no relationship between NO_2_ and CO and diabetes in all the groups. For every 10 *μ*g/m^3^ increase in PM_2.5_, the relative RR of diabetes was 1.163 (95% CI: 1.032-1.311) in females, while there was no statistical correlation in men. In the different age groups, PM_2.5_ was more strongly associated with diabetes in older people (≥75 years) than those aged under 75 years. For every 10 *μ*g/m^3^ increase in PM_2.5_, the RR of diabetes incidence increased by 1.140 (95% CI: 1.032-1.259). Exposure to PM_2.5_ was positively correlated with diabetes incidence in healthy weight and obese people. When the concentration of PM_2.5_ increased by 10 *μ*g/m^3^, the relative RR of obese and healthy-weight people was 1.135 (95% CI: 1.007-1.279) and 1.221 (95% CI: 1.042-1.431), respectively.

## 4. Discussion

Our study found that, in the population aged ≥60 years old from the Binhai New District in 2014-2017, there was a positive correlation between exposure to not only PM_2.5_ but also PM_10_ and diabetes incidence after multiple adjustments and between NO_2_, CO, and diabetes incidence in a single-pollutant model. Further stratification analysis found that the relationship between exposure to PM_2.5_ and diabetes was more robust in female subjects, subjects aged ≥75 years old, and obese people. In addition, meteorological factors were also found to be associated with the development of diabetes, and this association was nonlinear.

### 4.1. PM_2.5_

In the population aged 60 and over in the Tianjin Binhai New District, we found that PM_2.5_ was positively correlated with the incidence of diabetes in both the single-pollutant and the dual-pollutant models, which was consistent with a previous study conducted in Hong Kong, China [33]. In our multivariate model, PM_2.5_ increases by each interquartile range (53 g/m^3^) resulting in a relative RR of diabetes of 1.59 (95% CI: 1.11-2.27), which represented a 59% increase in the risk of diabetes. The Hong Kong cohort study used logistic regression and time-varying Cox regression models to assess the prevalence and risk of diabetes associated with PM_2.5_ among the elderly and found that after adjusting for potential individual and neighborhood confounders, the hazard ratio (HR) for diabetes was 1.15 (95% CI: 1.05-1.25) per interquartile range (3.2 *μ*g/m^3^) increase in PM_2.5_. Although this finding suggests a relationship between long-term exposures to PM_2.5_ and our study investigated short-term effects, the results in different regions of China were similar. Compared with our study, their results showed a weaker correlation between PM_2.5_ and diabetes. We consider that this might be due to differences in statistical methods and adjustment factors. Furthermore, the PM_2.5_ levels in our study were significantly higher than those measured in the Hong Kong study.

Another cohort study followed US veterans for 8.5 years and showed that there were approximately 32 million diabetic patients due to prolonged exposure to PM_2.5_ in 2016, accounting for 14% of global diabetes patients [34]. A study based on the SALIA cohort in Germany found that traffic-related air pollution, including NO_2_ and PM_2.5_, was associated with an increased risk of type 2 diabetes in women aged 54-55 years [35]. Similarly, a cohort study in Canada with a 30-year follow-up of women between the ages 40 and 59 described the relationship between PM_2.5_ and diabetes using Poisson regression models and found that for every 10 *μ*g/m^3^ increase in PM_2.5_, the risk of type 2 diabetes increased by 28% [36]. A meta-analysis, including 13 studies conducted in Europe and North America, reached the same conclusion. In this study, a combined relative RR of type 2 diabetes was 1.10 (95% CI: 1.02-1.18) for every 10 *μ*g/m^3^ increase in PM _2.5_ [37]. A large cohort study in Rome, Italy, used logistic and Cox regression models to assess the relationship between long-term exposure to PM, nitrogen oxides (NO_x_), ozone (O_3_), and the incidence of type 2 diabetes at individual levels. For every 20 *μ*g/m^3^ increase in NO_x_ and 10 *μ*g/m^3^ increase in O_3_, the hazard ratio (HR) for diabetes was found to be 1.01 (95% CI:1.00-1.02) and 1.02 (95% CI:1.00-1.03), respectively. However, no clear correlation was found between PM_2.5_, PM_10_, and diabetes incidence [38]. This discrepancy might be explained by substantial differences between the levels of air pollutants in Italy and China. The average concentrations of PM in our study area were about three times than that of those measured in Italy. Additionally, Italy is a developed country in Europe, and there might be economic and ethnic differences with China. Finally, our study was aimed at the elderly population, and the Italy study was conducted in people over the age of 35; therefore, the tolerance to air pollution of the population might be different.

### 4.2. PM_10_ and NO_2_

We found that exposure to PM_10_ and NO_2_ was positively correlated with the incidence of diabetes, similar to the results of previous studies [39, 40]. A population-based study in Germany conducted a random sampling of 45 to 72-year-old residents in highly urbanized areas and followed them for 5.1 years. The study concluded that PM_10_ was associated with an increased risk of diabetes, and the RR obtained with Poisson regression adjusting for sex, age, BMI, lifestyle factors, area and individual-level socioeconomic status, and city was 1.05 (95% CI: 1.00-1.10) [42]. A meta-analysis combined the results of ten cohort studies and concluded that every 10 *μ*g/m^3^ increase in PM_10_ and NO_2_ was connected with an increased risk of diabetes by 10% (95% CI: 22%-47%) and 11% (95% CI: 7%-16%), respectively [41]. However, a study from Tianjin, China, showed different results. It was found that among people aged between 45 and 64 years, the risk of diabetes increased by 1.16% for every 10 *μ*g/m^3^ increase of NO_2_, while PM_10_ and diabetes were not significantly correlated [42]. However, our results showed that in the single-pollutant model, both PM_10_ and NO_2_ were positively correlated with the onset of diabetes, and the correlation between NO_2_ and diabetes was stronger. The number of diabetic cases increased by 5.1% for every 10 *μ*g/m^3^ increase in NO_2_. Both studies performed short-term health effects on diabetes using time-series analysis and were conducted in Tianjin. However, our study was aimed at the Binhai New District, and the scope of that study was the urban area of Tianjin. Furthermore, the research periods were different. Comparing the average levels of air pollution, we found that the air pollution level in the Binhai New District from 2014 to 2017 was higher than that in the urban area of Tianjin in 2008-2011. Additionally, the study population was older, leading to a more vulnerable population being exposed to higher levels of air pollution, likely causing more significant adverse effects.

### 4.3. O_3,_ CO, and SO_2_

Our results suggested that there was no statistically significant association between O_3_ and diabetes in the elderly population of the Binhai New District. Similarly, a six-year study conducted among Mexican-Americans explored the adverse effects of air pollution on insulin resistance resulting in a similar conclusion [43]. The production of ozone requires the action of ultraviolet rays. When there is a high concentration of environmental PM, the ultraviolet rays of solar radiation will be scattered to some extent [44]. O_3_ was negatively correlated with the concentration of PM_2.5_ and PM_10_, indicating that O_3_ may cause the elution period of PM_2.5_ and PM_10_, thus failing to reflect the correlation between O_3_ and diabetes. However, in 45,231 African-American women from 56 regions of the United States, air pollution was distributed to individual levels through EPA Models-3/Community Multiscale Air Quality (CMAQ) predictions fused with ground measurements. Cox proportional hazards models were used to analyze this relationship. The HR per interquartile range increase of O_3_ was 1.18 (95% CI: 1.04-1.34) for the incidence of diabetes in adjusted models [45]. The discrepancy with our data may be due to different statistical methods used. We explored at the group level, while the American study was specific to individuals. Additionally, that study only involved women, while we conducted our study on the whole elderly population, which may have resulted in differences in performance between different populations exposed to O_3_. In addition to O_3_, we did not find a correlation between SO_2_ and CO and the incidence of diabetes. A large cohort study with a longer follow-up is required to confirm these data.

### 4.4. Possible Mechanism behind the Effect of Air Pollution on Diabetes Incidence

Research on the mechanisms behind the relationship between air pollutants and the increased incidence of diabetes mainly focused on PM. In both animal and human experiments, the levels of interleukin-6 (IL-6) and tumor necrosis factor-*α* (TNF-*α*) were elevated after exposure to PM [46–48]. Inflammatory markers are significantly associated with diabetes, and elevated levels of IL-6 can increase the risk of diabetes by 31% [49], indicating that air pollution can affect the development of diabetes through systemic inflammatory responses. Besides, animal experiments have shown that exposure to PM_2.5_ is associated with a decrease of insulin signaling in the liver, which inhibits the translocation of glucose transporter 4 (GLUT4), providing evidence that air pollution enhances insulin resistance in the liver [46, 50]. Moreover, exposure to PM_2.5_ also induces lipid deposition in the liver and reduces gluconeogenesis, inhibiting insulin receptor substrate 1- (IRS-1-) mediated signaling in the liver, which is associated with insulin resistance and abnormal glucose homeostasis [51].

### 4.5. Meteorological Factors

#### 4.5.1. Temperature

Our study found that high temperatures promoted diabetes, and a previous study using 14-year longitudinal data from the United States reached a similar conclusion. Using meta-regression, they determined the effect of mean annual temperature on diabetes incidence between 1996 and 2009 for each US state. Compared to cold years, the incidence of diabetes was higher in warm years, and the age-adjusted diabetes incidence increased by 0.314 per 1°C [22]. However, no previous research was conducted in China on the correlation of diabetes incidence and meteorological factors. The activity of brown adipose tissue (BAT) is negatively correlated with outdoor temperature. Increased fatty acid flow to BAT can lead to a compensatory increase in glucose transport to other metabolically active tissues and even weight loss [52–54]. Therefore, insulin sensitivity is improved at lower temperatures, which may explain the adverse effects of elevated temperature on diabetes.

#### 4.5.2. Humidity

We found that extreme moist or dry air was harmful to human health. Research in the Midwest of Africa supports our result, showing that the number of diabetes cases was highest in the wettest months and the hospital admission rate of diabetic patients was also slightly higher during the rainy season [52–54]. This may be related to the decline in body function and poor physiological response abilities in humid conditions. Instead of meteorological factors themselves, humidity also interacts with air pollutants, in turn affecting human health. In a previous study (conducted in Beijing, adjacent to Tianjin) on the relationship between air pollutants and meteorological factors, it was found that there was seasonal variability in the correlation between humidity and air pollution. Lower humidity was likely causing air pollution in spring and higher humidity likely contributed to air pollution in the other seasons, partly explaining why extreme humidity affects human health adversely [55].

#### 4.5.3. Sunshine

In our study, a reduction of sunshine duration increased the risk of diabetes. The only other study that examined the association between sunshine and diabetes incidence was a prospective cohort study of women aged 25 to 64 years in southern Sweden. The average follow-up time was 11 years, and data were analyzed by logistic regression analysis. It was found that, compared to women without activity habits, women with sun exposure habits had a lower risk of developing type 2 diabetes [23]. Solar radiation is the primary source of vitamin D. Vitamin D appears to play a role in pancreatic diseases, including type 1 and type 2 diabetes [56]. It can affect the function of beta cells and influence insulin sensitivity. Furthermore, it can reduce systemic inflammatory responses by controlling blood pressure, reducing peripheral vascular resistance, or acting as an immunomodulator to prevent excessive production of inflammatory cytokines [57–59]. Therefore, sunshine may prevent the development of type 2 diabetes by increasing serum 25-(OH)2-D3 levels.

#### 4.5.4. Atmospheric Pressure

We found that low air pressure was associated with a higher diabetes incidence. No previous research investigated the impact of atmospheric pressure on diabetes incidence. However, a multicenter study showed that increased atmospheric pressure was beneficial to reduce mortality [60]. In addition, it was previously found that the concentration of air pollutants is affected by the domestic trans-boundary impacts (TBI), which contributed to 27% of the total PM_2.5_ in China [61]. At lower atmospheric pressure, between 900 hPa and 850 hPa, wind can more easily transport air pollutants between different regions, aggravating the adverse effects of air pollution. What is more, air pollution is relatively severe when atmospheric pressure is lower, partly affecting the relationship between low air pressure and diabetes.

#### 4.5.5. Precipitation

We found that precipitation might promote diabetes within a specific range, consistent with previous findings from studies conducted in the capital of Cameroon, a Midwestern African country [62]. However, in our study, when the precipitation exceeded the 99th percentile (60 mm), it was a protective factor within 20 days, and it showed adverse effect on diabetes after 23 days. Given this result, we consider that precipitation was greater than 60 mm only in 2 out of the 1461 days, and deviations might be caused by these extreme values, as rainstorms could reduce air pollutants on the day of measurement [63]. Therefore, the combined effect makes the number of onset diabetes decline in the short term.

#### 4.5.6. Wind Speed

A study in Beijing showed that the effect of wind speed on air quality differed between seasons. When the wind speed was less than 1 m/s, the probability of air pollution in autumn and winter was more than 68.06%, while when the wind speed was 2 m/s in spring and summer, the probability of pollution reached the maximum [55]. This is in line with our data. We showed that the number of people with diabetes increased when wind speed was under 2 m/s. We hypothesize this may be related to the slower dissipation of air pollutants at low wind speed.

### 4.6. Subgroup Analysis

To adjust for possible confounding factors, we conducted a subgroup analysis based on gender, age, and BMI. Analysis by gender showed that there was a more significant association between PM_2.5_ and diabetes incidence in women (RR: 1.12; 95% CI: 1.02-1.22). The Hong Kong study mentioned earlier reached the same conclusion [55]. That study involved 66,820 adults aged 65 years or older and stratified analyses by sex. They showed that the associations between PM_2.5_ exposure and prevalence and incidence of type 2 diabetes mellitus were only statistically significant in women (RR: 1.07; 95% CI: 1.01-1.13). Additionally, a meta-analysis combining the results of four studies in Europe or the United States found that women tended to be affected more than men, with a relative risk of 1.14 (95% CI: 1.03-1.26) in women and 1.04 in men (95% CI: 0.93-1.17) [37]. It seems the response intensity of men and women to air pollution is different, but the specific relationship and the mechanism behind it remain unclear. This may be due to gender-related biological differences, such as hormones and body size, diet, and activity patterns. Women's airway diameters are different from those in men, which leads to an easier deposition of PM_2.5_ in female airways [64]. In the subgroup analysis, we also found that the population aged ≥75 years (RR: 1.14; 95% CI: 1.03-1.31) was more susceptible to PM_2.5_ than the younger population (RR: 1.16; 95% CI: 1.03-1.31). A case-crossover study conducted in Anshan, China, explored the relationship between air pollution and daily mortality and also found that people aged ≥75 years were more susceptible to air pollutants, supporting our conclusion [65]. This may be attributed to the gradual decline of physiological function, as well as the poorer physical reserves and stress response of older people [66]. The immune system of the elderly is weakened, and aging has been associated with subclinical systemic inflammation. Therefore, older people are prone to systemic inflammation when they are persistently exposed to air pollutants [67]. We found a positive correlation between PM_2.5_ and diabetes incidence in healthy weight and obese people. A cohort of 28,831 nurses in Denmark found that PM_2.5_ was most strongly associated with diabetes in obese people, with a hazard risk of 1.25 (95% CI:1.06-1.47) [68]. Exposure to air pollutants could promote the occurrence and progression of inflammatory reactions, and obesity is associated with higher levels of systemic inflammation, making obese people more susceptible to air pollution [48, 69].

### 4.7. Limitations and Strengths

First of all, diabetes is a chronic disease, and it is challenging to determine the exact day of incidence, so we took the diagnosis date instead. Secondly, due to limitations in the data available and technical constraints, we could not accurately correlate air pollutant concentrations to individuals. Therefore, we could only study patients at the group level. Third, there were many potential confounding factors, such as noise, and their interaction with air pollution has not been widely evaluated. However, the effects of meteorological factors and long-term trends were considered in this study, and subgroup analyses were performed based on age, gender, and BMI, to ensure the results could explain the association between diabetes and air pollution to some extent. Another limitation is that we only obtained data about outdoor air pollutants, while indoor air pollution concentrations, as well as those in some working environments, are much higher than outdoors [70, 71]. Another issue is that air pollutants are complex mixtures. It consists of variable physical properties or chemical compositions, and we could not analyze the single components. However, the single and multivariate models we used are accepted widely, making analysis feasible and comparable.

This is the first study in China into the possible association between air pollutants in the coastal area and the risk of diabetes in the elderly population and fills the gap of knowledge of the impact of Chinese meteorological factors on the risk of diabetes.

## 5. Conclusion

We found a positive correlation between air pollutants, such as PM_2.5_, and diabetes risk, consistent with previous studies. This finding may have an impact on strategies to reduce diabetes risk. Meteorological factors were also found to play an essential role in the occurrence, development, and dissipation of air pollution and can also directly affect diabetes. However, due to the limitations of this study, more advanced air pollutant estimation techniques and statistical methods are needed to explore in a wider geographical area and a larger population.

## Figures and Tables

**Table 1:** The population characteristics at baseline.

	Median (P25, P75) (*n* = 190453)
Age (years)	68 (64, 72)
Weight (kg)	66 (60, 73)
Height (cm)	165 (159, 170)
BMI (kg/m^2)^	24.0 (22.6 ,26.2)
Waistline (cm)	84 (79, 90)
TG (mmol/L)	1.30 (1.01, 1.71)
TC (mmol/L)	5.04 (4.43, 5.70)
BUN (mmol/L)	5.40 (4.58, 6.35)
Scr (*μ*mol/L)	73.0 (60.8, 86.0)
TB (*μ*mol/L)	12.7 (10.0, 16.2)
AST (U/L)	21.0 (17.0, 26.0)
ALT (U/L)	19.0 (14.0, 25.0)
FBG (mmol/L)	5.30 (4.91, 5.70)
Hemoglobin (g/L)	137 (128, 147)

	*N* (%) (*n* = 190453)
Sex, male	91237 (47.91)
Physical activity	
Never	53418 (28.05)
Once a week	10587 (5.56)
More than once a week	32531 (17.08)
Every day	93914 (49.31)
Vegetables/meat ratio	
<2	2284 (1.20)
=2	181859 (95.49)
>2	6310 (3.31)
Smoking status	
Never	141345 (74.22)
Current	37903 (19.90)
Former	11205 (5.88)

BMI: body mass index; TC: total cholesterol; TG: triglyceride; BUN: blood urea nitrogen; Scr: serum creatinine; TB: total bilirubin; ALT: glutamic-pyruvic transaminase; AST: glutamic oxalacetic transaminase; FBG: fasting blood glucose.

**Table 2:** Air pollutants in Binhai New Area during 2014 to 2017.

	Min	25% quartile	Median	75% quartile	Max	Mean	Standard deviation	Interquartile range
PM_2.5_ (*μ*g/m^3^)	10.68	36.38	56.55	89.10	343.33	70.56	49.22	52.72
PM_10_ (*μ*g/m^3^)	11.11	64.51	96.24	142.87	975.75	96.24	72.60	78.36
NO_2_ (*μ*g/m^3^)	9.18	30.44	43.78	61.91	177.77	48.20	23.50	31.47
SO_2_ (*μ*g/m^3^)	1.98	10.64	16.94	29.95	161.48	23.55	19.69	19.31
O_3_ (*μ*g/m^3^)	2.57	26.39	46.68	75.25	174.43	53.18	33.94	48.86
CO (mg/m^3^)	0.27	0.91	1.25	1.71	9.23	1.42	0.78	0.80

**Table 3:** Meteorological factors in Binhai New Area during 2014 to 2017.

	Min	25% quartile	Median	75% quartile	Max	Mean	Standard deviation	Interquartile range
Average temperature (°C)	-14.50	3.45	15.40	24.25	33.30	14.27	10.84	20.80
Maximum temperature (°C)	-11.30	7.80	20.30	28.50	39.33	18.58	11.07	20.70
Humidity (%)	13	43	57	72	99	57	18.19	29
Precipitation (mm)	0.00	0.00	0.00	0.00	148.30	1.52	7.14	0.00
Sunshine (h)	0.00	4.10	8.00	10.00	13.60	6.98	3.94	5.90
Average pressure (hPa)	994	1008	1017	1025	1043	1017	10	17
Average wind speed (m/s)	0.50	1.80	2.30	3.00	9.40	2.47	0.95	1.20

**Table 4:** Spearman correlation analysis between air pollutants and meteorological variables.

	Number	PM_2.5_	PM_10_	SO_2_	O_3_	NO_2_	CO	Humidity	Temperature	Precipitation	Sunshine	Pressure
PM_2.5_	.039											
PM_10_	.078^∗∗^	.897^∗∗^										
SO_2_	.024	.548^∗∗^	.581^∗∗^									
O_3_	.148^∗∗^	-.228^∗∗^	-.228^∗∗^	-.449^∗∗^								
NO_2_	.123^∗∗^	.611^∗∗^	.611^∗∗^	.721^∗∗^	-.531^∗∗^							
CO	.008	.657^∗∗^	.590^∗∗^	.734^∗∗^	-.448^∗∗^	.688^∗∗^						
Humidity	-.122^∗∗^	.265^∗∗^	.032	-.161^∗∗^	.012	-.082^∗∗^	.185^∗∗^					
Temperature	-.031	-.122^∗∗^	-.162^∗∗^	-.509^∗∗^	.769^∗∗^	-.482^∗∗^	-.400^∗∗^	.229^∗∗^				
Precipitation	-.058^∗^	-.151^∗∗^	-.259^∗∗^	-.302^∗∗^	.089^∗∗^	-.271^∗∗^	-.118^∗∗^	.425^∗∗^	.151^∗∗^			
Sunshine	.130^∗∗^	-.271^∗∗^	-.135^∗∗^	-.142^∗∗^	.425^∗∗^	-.216^∗∗^	-.331^∗∗^	-.539^∗∗^	.300^∗∗^	-.335^∗∗^		
Pressure	-.033	.043	.059^∗^	.455^∗∗^	-.714^∗∗^	.438^∗∗^	.341^∗∗^	-.234^∗∗^	-.886^∗∗^	-.216^∗∗^	-.252^∗∗^	
Wind speed	.101^∗∗^	-.273^∗∗^	-.145^∗∗^	-.201^∗∗^	.221^∗∗^	-.371^∗∗^	-.311^∗∗^	-.352^∗∗^	-.004	-.008	.240^∗∗^	-.070^∗∗^

**Table 5:** Fitting results of single and double air pollutant model.

Model	Risk ratio (95% CI)	
	Increase 10 *μ*g/m^3^ or 1 mg/m^3^	Increase the interquartile range
*PM_2.5_*		
PM_2.5_	1.026 (1.011-1.040)^∗^	1.144 (1.062-1.233)^∗^
PM_2.5_ + PM_10_	0.994 (0.972-1.017)	0.971 (0.862-1.093)
PM_2.5_ + NO_2_	1.020 (1.001-1.038)^∗^	1.109 (1.007-1.222)^∗^
PM_2.5_ + SO_2_	1.033 (1.016-1.050)^∗^	1.189 (1.090-1.297)^∗^
PM_2.5_ + CO	1.022 (1.005-1.039)^∗^	1.121 (1.026-1.225)^∗^
PM_2.5_ + O_3_	1.026 (1.012-1.041)^∗^	1.147 (1.064-1.236)^∗^
*PM_10_*		
PM_10_	1.019 (1.012-1.026)^∗^	1.157 (1.093-1.225)^∗^
PM_10_ + PM_2.5_	1.021 (1.010-1.033)^∗^	1.178 (1.077-1.289)^∗^
PM_10_ + NO_2_	1.017 (1.009-1.025)^∗^	1.142 (1.072-1.216)^∗^
PM_10_ + SO_2_	1.021 (1.013-1.029)^∗^	1.179 (1.109-1.252)^∗^
PM_10_ + CO	1.018 (1.010-1.026)^∗^	1.146 (1.077-1.218)^∗^
PM_10_ + O_3_	1.019 (1.012-1.027)^∗^	1.158 (1.094-1.226)^∗^
*NO_2_*		
NO_2_	1.051 (1.019-1.083)^∗^	1.171 (1.063-1.290)^∗^
NO_2_ + PM_2.5_	1.023 (0.984-1.063)	1.075 (0.949-1.217)
NO_2_ + PM_10_	1.022 (0.989-1.057)	1.073 (0.966-1.193)
NO_2_ + SO_2_	1.078 (1.037-1.120)^∗^	1.270 (1.122-1.438)^∗^
NO_2_ + O_3_	1.051 (1.019-1.083)^∗^	1.171 (1.062-1.291)^∗^
NO_2_ + CO	1.032 (0.995-1.071)	1.107 (0.984-1.244)
*CO*		
CO	1.156 (1.058-1.264)^∗^	—
CO + NO_2_	1.097 (0.984-1.224)	—
CO + PM_2.5_	1.065 (0.958-1.185)	—
CO + PM_10_	1.066 (0.968-1.174)	—
CO + SO_2_	1.195 (1.074-1.329)^∗^	—
CO + O_3_	1.160 (1.061-1.269)^∗^	—

**Table 6:** Results of subgroup analysis based on sex, age, and BMI.

	T2DM Number	PM_2.5_	NO_2_ RR (95% CI)	CO
*Sex*				
Male	3405	1.070 (0.988-1.157)	1.006 (0.860-1.177)	0.943 (0.603-1.473)
Female	4180	1.163 (1.032-1.311)^∗^	1.044 (0.846-1.289)	0.837 (0.508-1.380)
*Age*				
<75	6214	1.109 (1.031-1.193)^∗^	1.071 (0.894-1.283)	0.805 (0.528-1.228)
≥75	1371	1.140 (1.032-1.259)^∗^	0.803 (0.633-1.018)	1.167 (0.626-2.175)
*BMI*				
Low weight (<18.5)	51	0.960 (0.542-1.700)	0.477 (0.163-1.402)	2.691 (0.129-55.997)
Normal (18.5-23.99)	2138	1.221 (1.042-1.431)^∗^	0.829 (0.564-1.219)	0.593 (0.242-1.451)
Overweight (24-27.99)	3715	1.074 (0.986-1.169)	1.062 (0.866-1.302)	0.810 (0.481-1.364)
Obesity (≥28)	1698	1.135 (1.007-1.279)^∗^	0.811 (0.614-1.070)	1.270 (0.625-2.578)
Total	7585	1.092 (1.021-1.168)^∗^	1.053 (0.889-1.247)	0.851 (0.574-1.261)

## Data Availability

The data are included in the article.

## Supplementary Materials

Supplementary Materials The GAM for meteorological factors was partly same as that for air pollution and is shown in the Supplementary Materials.
