# Supplementary material for: Risk Analysis of Air Pollution and Meteorological Factors Affecting the Incidence of Diabetes in the Elderly Population in Northern China
Source: J Diabetes Res. 2020 Oct 20;2020:3673980. doi: 10.1155/2020/3673980 (PMC7593725; doi:10.1155/2020/3673980)
Supplement: Supplementary Materials — The GAM for meteorological factors was partly same as that for air pollution and is shown in the Supplementary Materials. [file 3673980.f1.docx]

**Supplementary Materials**

the GAM for meteorological factors is as following:

“LogE（Yt）= cb (M, l) + DOW + ns（time, df） （2）

+ intercept

Where Yt represented the expected number of diabetes cases; cb (M, l) denoted the cross-basis function for the mainly studied meteorological factor, it is non-linear, so that without coefficient; ns was a natural cubic spline function, df was its degree of freedom; time was a date variable used to control time trends and seasonal fluctuations; DOW represented the day of the week, controlling the natural fluctuations of the number of people with diabetes in a week.”
